# Physics-Informed Neural Network-Based Elevator Degradation Diagnosis and Early Warning

**DOI:** 10.3390/s26123718

**Published:** 2026-06-11

**Authors:** Ren Li, Gang Xiao, Yuanming Zhang, Yaxing Ren, Fangfang Yao, Xiaoying Ru, Zhenhao Li

**Affiliations:** 1 Zhejiang University of Technology, Gongshu District, Hangzhou 310014, China; 122224120042@zjut.edu.cn (R.L.);; 2 Sicher Elevator Co., Ltd., Nanxun District, Huzhou 313013, China

**Keywords:** physics-informed neural network, elevator health monitoring, early fault warning, predictive maintenance, degradation assessment

## Abstract

With the continuous growth of urban building density and elevator deployment, the reliability, maintenance, and degradation risk warning of elevator systems have attracted increasing attention. Conventional monitoring methods based on fixed thresholds or rule logic are easy to implement, but they often fail to identify progressive degradation and are sensitive to complex operating conditions and measurement noise. This paper proposes a physics-informed neural network (PINN)-based method for elevator health monitoring and early warning. First, multi-sensor data are processed through time alignment and feature reconstruction, and a dual-path acceleration estimation method is introduced to improve the stability of dynamic state calculation. Second, a simplified traction elevator dynamic model considering load variation, motor drive, and mechanical resistance is embedded into PINN training to identify hidden parameters. Electrical and dynamic residual indicators are then constructed to characterise system condition from different physical perspectives. Finally, a time-accumulated risk model combining anomaly magnitude and persistence duration is developed to detect progressive degradation trends. Results show stable parameter convergence and effective condition assessment. The proposed approach detects degradation trends earlier than conventional threshold-based monitoring methods and reduces false alarms caused by transient disturbances. It provides an interpretable and practical solution for predictive maintenance and intelligent operation of elevator systems.

## 1. Introduction

Elevator systems are an essential component of modern urban infrastructure and provide indispensable vertical transportation in high-rise buildings. With the acceleration of urbanisation and the increasing number of super-tall buildings, both the operating frequency and system complexity of elevators have risen significantly, leading to higher requirements for safety and reliability. In typical commercial and residential scenarios, elevators perform frequent start–stop operations under varying load conditions every day. Once a fault occurs, it may not only interrupt service but also cause passenger entrapment or even safety incidents. Therefore, continuous health monitoring and fault warning are of great importance for ensuring operational safety and reducing maintenance costs [1].

Traditional elevator fault detection methods mainly rely on fixed thresholds and rule-based logic. In practice, key operating parameters such as speed, voltage, current, and door-lock status are assigned predefined limits, and alarms or protective actions are triggered when signals exceed these ranges [2]. These methods are widely adopted in industrial systems because of their fast response, simple implementation, and high reliability. However, they are essentially static decision mechanisms and are difficult to adapt to complex and varying operating conditions. They are also sensitive to noise, which may lead to false alarms or missed detections. In addition, elevator systems exhibit strong electromechanical coupling, where dynamic behaviour is jointly influenced by load variation, friction condition, and motor characteristics. Conventional rule-based approaches are unable to effectively capture such complex dynamic interactions, especially for progressive faults such as guide rail wear or reduced motor efficiency.

With the development of the industrial Internet of Things and data analytics, data-driven intelligent diagnosis methods have gradually become a major research topic [3,4]. Various machine learning and deep learning approaches have been explored in academia and industry [5], including fault tree analysis [6], support vector machine models [7,8], long short-term memory networks [9,10], and convolutional neural networks for fault classification and prediction [5]. These methods can automatically extract features from historical data and provide strong nonlinear modelling capability, achieving promising results in bearing fault diagnosis, motor anomaly detection, and vibration analysis [11].

Although data-driven methods perform well in pattern recognition, several challenges remain for practical elevator applications. First, model training usually requires large volumes of labelled data, particularly fault samples. However, faults in real elevator systems occur infrequently, are diverse in type, and are often incompletely recorded, resulting in limited high-quality fault datasets. Second, purely data-driven models often lack physical interpretability, and their outputs cannot be directly linked to actual equipment mechanisms, which limits user confidence in maintenance decisions. Third, elevator operating conditions are highly non-stationary. Variations in load, floor travel range, direction of motion, and usage period may all change the data distribution and reduce model generalisation capability [12]. Finally, many critical health-related variables, such as friction resistance, drive force deviation, and equivalent mass variation, cannot be directly measured, making accurate degradation assessment difficult when only observed signals are used.

To improve interpretability and generalisation, physics-informed neural networks (PINNs) have attracted increasing attention in recent years [13]. By embedding physical constraints such as differential equations or conservation laws into the training process, PINNs enable neural networks to fit measurement data while satisfying system physics, thereby achieving reliable modelling under limited data conditions [14]. This approach has been applied in fluid mechanics, power systems, and structural health monitoring, showing strong generalisation performance [15]. For elevators and similar electromechanical systems, PINNs provide an effective way to combine dynamic models with operational data. However, existing PINN studies still face several limitations in engineering applications. Most methods construct residual functions from a single observed variable and do not fully exploit complementary information from multiple physical quantities, which restricts fault discrimination capability. In addition, PINNs are commonly designed for offline modelling and high-accuracy computation, often with considerable computational cost, and lack lightweight designs for real-time monitoring scenarios [16].

For elevator systems, PINNs are naturally suitable. On one hand, elevator operation follows clear dynamic and drive mechanisms that can be described by load balance relations, motor input–output relationships, and kinematic equations. On the other hand, many important degradation indicators in practice cannot be directly measured, such as increased friction, reduced drive efficiency, or shifted load balance. These hidden parameters are closely related to system health. Therefore, integrating operational data with physical models through PINNs to estimate hidden parameters online and construct health indicators offers a feasible pathway for degradation risk warning.

Motivated by these considerations, this paper proposes a PINN-based health monitoring and degradation risk warning method for traction elevator systems. First, a simplified dynamic model incorporating load variation, motor drive, and mechanical resistance is established, and a PINN parameter identification framework suitable for field data is developed. Second, dual-residual health indicators based on electrical residuals and dynamic residuals are designed to characterise system condition from different physical perspectives. Third, a time-accumulated risk model integrating anomaly magnitude and persistence duration is proposed to achieve early identification of progressive degradation. Finally, practical operational data are used to validate the effectiveness of the proposed method in parameter estimation, health assessment, and early warning.

The main contributions of this work are summarised as follows:

1. A simplified electromechanical coupling model for traction elevators is developed and embedded into the PINN training process to identify key hidden parameters.
2. Dual-residual health indicators based on electrical-domain and mechanical-domain information are proposed to improve the robustness and interpretability of condition assessment.
3. A time-accumulated risk model considering anomaly persistence is established for early warning of degradation trends and continuous health indicator generation.
4. The proposed method is validated using real operational data and compared with conventional threshold-based monitoring approaches, demonstrating its engineering feasibility and early warning capability for predictive maintenance of elevator systems.

## 2. Dynamic Model of the Traction Elevator System

To realise physics-constrained fault risk warning for elevator systems, it is first necessary to establish a mathematical model that can represent the operating characteristics of the system and integrate it with a PINN. In this way, physical constraints can be introduced into the data-driven modelling process, thereby improving model interpretability and generalisation capability.

**Figure 1:**
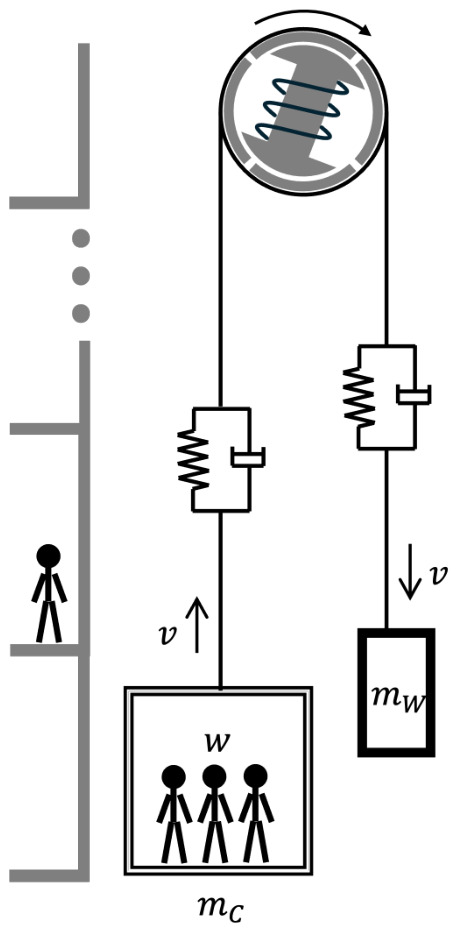
Schematic diagram of the traction elevator system.

Traction elevator systems are currently the most widely used type of elevator. They mainly consist of the elevator car, counterweight, traction machine, steel ropes, and guide rail system [17], as shown in Figure 1. The traction machine drives the traction sheave, causing rope displacement and thereby moving the car and counterweight vertically along the guide rails. During operation, the car and counterweight form a mass-balancing system, while the motor mainly compensates for the unbalanced force caused by variations in cabin load and system friction. Therefore, the dynamic characteristics of the elevator system are closely related to changes in cabin load.

In engineering design, the counterweight mass is typically selected as a certain proportion of the elevator car mass and rated load. A common design practice is to set the counterweight equal to the car mass plus approximately 50% of the rated load, so as to reduce motor driving demand and energy consumption [17]. This configuration allows the elevator to achieve optimal operating performance near the half-load condition. However, in practical operation, the number of passengers continuously changes, and the cabin load varies over time, resulting in system mass imbalance and affecting the operating characteristics of the elevator. Therefore, establishing a dynamic model that incorporates load variation is of great significance for subsequent degradation diagnosis and risk assessment.

In addition, the traction machine is commonly driven by an AC motor, such as a permanent magnet synchronous motor or an induction motor, and is equipped with a mechanical brake as a safety protection device. During upward travel, the motor mainly provides driving torque to overcome the gravitational imbalance and friction resistance. During downward travel, some elevator systems rely partly on gravity, while the braking system regulates the travelling speed. Owing to these different driving mechanisms, elevators exhibit clear electrical and mechanical asymmetry during upward and downward motion, which should be considered in motor modelling and degradation diagnosis.

### 2.1. Electrical Model of the Traction Motor

The traction machine is commonly driven by an AC motor. Its complete representation is usually based on the *d*-*q* axis model. However, such a model requires variables that are difficult to obtain directly, such as current and flux linkage, and it also involves complex control system modelling. Therefore, it is not suitable for online parameter identification using operational data. To enable practical engineering implementation, a simplified motor model is adopted in this study to describe the relationship among voltage, speed, and driving force. In this model, the motor output driving force is assumed to be proportional to the current, while the motor voltage consists of the base voltage, resistive voltage drops, and back electromotive force. The proposed model captures the essential driving characteristics of the motor while avoiding the difficulties associated with detailed motor control modelling. The simplified steady-state model used in this paper is given as follows:

(1) $$V\left(t\right)=V_{0}+RI\left(t\right)+K_{e}v\left(t\right)$$

(2) $$F_{mot}\left(t\right)=K_{t}I\left(t\right)$$

where $V_{0}$ denotes the base voltage associated with no-load operation or excitation. *R* is the equivalent resistance. $K_{e}$ denotes the equivalent back electromotive force (EMF) coefficient, which represents the relationship between elevator velocity and induced voltage in the traction motor. $K_{t}$ denotes the equivalent motor torque coefficient, describing the conversion relationship between motor current and traction force output, and I(t) is the motor current.

Therefore, the voltage response differs between upward and downward travel. By eliminating the current term I(t), the following expression can be obtained:

(3) $$V\left(t\right)=V_{0}+\frac{F_{mot}\left(t\right)}{K_{IR}}+K_{e}v\left(t\right)$$

where

(4) $$K_{IR}=\frac{K_{t}}{R}$$

which represents the equivalent electromechanical driving coefficient combining motor torque conversion and circuit resistance characteristics.

### 2.2. Elevator Dynamic Model

In existing studies, elevator systems are commonly described using multi-body dynamic models that consider rope elasticity, pulley dynamics, and structural vibration [18]. However, such models are computationally complex and require a large number of structural parameters that are difficult to obtain in practice. Therefore, they are not well suited for online monitoring and real-time fault warning applications. To reduce model complexity and improve the feasibility of parameter identification, an equivalent single-degree-of-freedom model is adopted in this study, in which the elevator system is simplified as a one-dimensional vertical motion system. $m_{eq}$ represents the lumped equivalent moving mass of the elevator system, including the influence of car mass, counterweight imbalance, and passenger load:

(5) $$m_{eq}\left(t\right)=M_{car}+M_{L}w\left(t\right)$$

where $M_{car}$ represents the mass difference between the elevator car and the counterweight, $M_{L}$ is the rated load, and w(t) denotes the normalised load ratio. This formulation combines the elevator car, counterweight, and load variation into a unified equivalent mass. The passenger load information is obtained from the elevator load-weighing sensor installed beneath the car platform. The sensor measures the total load inside the elevator car, including passengers and carried objects, and outputs a normalised load ratio relative to the rated load.

Since the cabin load varies with time, the equivalent mass is treated as a time-varying parameter. The model can capture the influence of load variation on the dynamic characteristics of the elevator system while avoiding uncertainties introduced by complex structural parameter modelling.

Based on Newton’s second law, the driving force of the elevator system can be expressed as the sum of the equivalent mass multiplied by acceleration and the system friction force. The dynamic equation of the system is given by:

(6) $$F_{dyn}\left(t\right)=m_{eq}\left(t\right)g+m_{total}a\left(t\right)+F_{brake}\left(t\right)$$

where a(t) is the acceleration of the elevator car, *g* is the gravitational acceleration constant, $m_{total}$ is the total mass of the elevator car, counterweight and load, and $F_{brake}$ is the equivalent braking force.

In practical elevator systems, the operating states of the motor differ during upward and downward travel. For elevators equipped with energy regeneration systems, the electrical characteristics in both directions are relatively symmetric. However, in conventional traction systems, downward travel often relies on the braking system for speed regulation, resulting in different electrical responses. During operation, friction mainly arises from guide rail contact, bearing resistance, and transmission losses in the traction system. In this study, an equivalent brake system model is adopted to combine different friction effects into an equivalent damping term, thereby reducing model complexity and improving the stability of parameter identification. The braking force is commonly represented by a combined model consisting of Coulomb friction, viscous friction, and braking system resistance:

(7) $$F_{brake}\left(t\right)=F_{b}\left(t\right)+\mu v\left(t\right)$$

where $F_{b}\left(t\right)$ is the lumped braking force of the elevator mechanical system, and μ is the equivalent viscous friction coefficient. The model considers both inertial force and friction resistance, where the friction term is expressed in a velocity-dependent form to describe the combined damping effects of the guide rails, bearings, and mechanical transmission system.

The proposed model jointly considers load variation, motor drive, mechanical friction, and braking effects. While maintaining relatively low complexity, it can effectively capture the main dynamic characteristics of elevator operation and provides a physical foundation for subsequent PINN-based parameter identification and risk warning.

## 3. Unknown Parameter Estimation Method Based on PINN

### 3.1. Basic Principle of PINN

PINNs are a machine learning approach that incorporates known physical laws into the neural network training process. Unlike conventional neural networks that rely solely on data fitting, PINNs learn data features while simultaneously introducing physical constraints, so that the model outputs are consistent not only with observed data but also with fundamental physical principles.

A typical PINN mainly consists of four components: a neural network model, physical constraints, a loss function, and an optimisation algorithm. The neural network serves as a nonlinear function approximation to learn complex mappings between inputs and outputs. Given input variables *x*, such as time, speed, load, voltage, and other measurable signals, the network produces the predicted output *y*. The key feature of PINN is the incorporation of known physical laws into the training process as constraints. These physical laws may originate from dynamic equations, electrical equations, kinematic relationships, conservation laws, or empirical models, enabling the model outputs to satisfy fundamental system behaviour.

The training objective of PINN is usually formulated through a combined loss function:

(8) $$L_{PINN}=\lambda_{d}L_{data}+\lambda_{p}L_{physics}$$

where $L_{data}$ denotes the data fitting loss used to measure the error between model predictions and actual measurements, $L_{physics}$ denotes the physics-based loss used to quantify the violation of physical equations, and $\lambda_{d}$ and $\lambda_{p}$ are weighting coefficients used to balance data fitting and physical constraints.

Through this loss function, the model not only seeks prediction accuracy but also avoids generating results that violate physical laws. Finally, optimisation algorithms are employed to iteratively update the neural network parameters together with the unknown physical parameters to be identified, so that the total loss gradually decreases until convergence is achieved.

Compared with conventional purely data-driven methods, PINN offers several advantages. First, it reduces the dependence on large datasets, since physical knowledge can support training even when samples are limited. Second, it improves generalisation capability and is often more stable than black-box models under unseen operating conditions. Third, it provides stronger interpretability, as model parameters often correspond to real physical quantities. Fourth, it can estimate unmeasurable variables, such as friction coefficients, driving force, and internal hidden states.

### 3.2. PINN Modelling of the Elevator System

For elevator systems, many key indicators, such as increased mechanical friction, load imbalance, and motor performance degradation, cannot be directly measured, while available operational data usually include only limited signals such as speed, voltage, current, and weight. PINN can utilise these measurable data together with the elevator dynamic and motor models to estimate unmeasurable parameters online, which can then be used for condition monitoring and degradation risk warning. Therefore, PINN provides a practical solution for intelligent elevator maintenance that combines physical consistency with engineering applicability.

**Figure 2:**
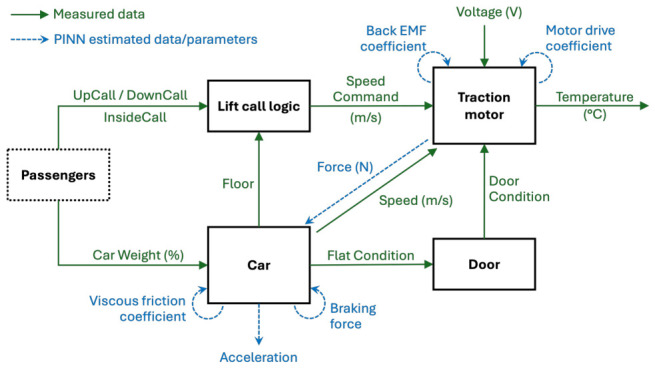
Elevator operational signal acquisition and data relationships.

Based on the dynamic model and drive model established in Section 2, a PINN-based parameter identification framework for traction elevator systems is developed in this study. Table 1 lists the operating signals that are commonly available from practical elevator control cabinets. These variables form the data foundation for the proposed PINN modelling and health monitoring framework. By analysing the existing sensor data from the control cabinet, the mapping relationship between measured variables and PINN-estimated variables can be established, as illustrated in Figure 2. The figure shows the physical relationships among key variables during elevator operation, including motor drive, car motion, and load variation, while distinguishing directly measurable variables from hidden variables that need to be inferred through the PINN model. This relationship provides an important basis for PINN modelling.

#### 3.2.1. Lightweight Neural Network Architecture

Unlike conventional PINN implementations that rely primarily on deep neural networks to approximate system dynamics, the proposed framework adopts a hybrid architecture that combines a physics-based elevator model with a lightweight residual neural network.

The physical model captures the dominant dynamic and electrical behaviour of the traction elevator through interpretable parameters, including the equivalent mass imbalance, friction coefficient, motor driving coefficient, and back-EMF coefficient. A lightweight feed-forward neural network is then introduced to compensate for modelling inaccuracies and unmodelled nonlinear effects.

The neural network consists of an input layer with three inputs (signed velocity, load ratio, and acceleration), two hidden layers with 16 neurons per layer, and an output layer with two neurons corresponding to voltage and force correction terms. Hyperbolic tangent (Tanh) activation functions are employed in the hidden layers.

The resulting network contains approximately 370 trainable neural network parameters, which is significantly smaller than conventional PINN architectures reported in the literature. Since the dominant system behaviour is represented by embedded physical equations, the neural network only needs to learn residual modelling errors. This lightweight design improves interpretability, and reduces the risk of overfitting under limited training data conditions.

#### 3.2.2. Physics-Informed Elevator PINN Training

In this study, the elevator dynamic model and motor model are embedded into the PINN training process as physical constraints. The network inputs are elevator operating state variables, such as speed, load, and acceleration, while the outputs are the predicted voltage and driving force. By minimising the error between predictions and measured data while satisfying the dynamic and motor model constraints, system parameter identification can be achieved.

This modelling approach enables the learning of normal operating characteristics even in the absence of fault data, and fault warning can be realised through residual analysis. In addition, since the model parameters have physical meaning, parameter variations can directly reflect changes in system condition, thereby further improving fault diagnosis capability.

To enhance practical applicability, several reasonable simplifications are introduced in the modelling process. First, rope elasticity and slip effects are neglected to reduce model complexity. Second, an equivalent friction model is adopted to represent the aggregated damping effects arising from guide rails, bearings, traction components, and other mechanical transmission elements. In addition, high-frequency motor dynamics are ignored, and only steady-state electrical relationships are considered. Finally, equivalent parameter modelling is employed to represent the dominant electromechanical behaviour of the elevator system using a small number of lumped parameters, thereby reducing the influence of quantities that are difficult to measure directly. The term ’equivalent’ in this study therefore refers to a lumped-parameter representation obtained through engineering simplification rather than a strict physical or mathematical equivalence.

These simplifications make the model more suitable for online monitoring and fault trend analysis while maintaining good physical consistency. Although the model is not intended for high-precision control design or high-frequency vibration analysis, it is well suited for elevator fault warning and condition assessment, and it provides a theoretical foundation for subsequent health monitoring and degradation risk warning.

### 3.3. Parameter Identification and Multi-Residual Loss Function Design

In the PINN model, the input variables are elevator operating state variables, including speed, load, and acceleration. These variables can reflect the dynamic state of the elevator system and are used as inputs to the neural network:

(9) x(t)=[v(t),w(t),a(t)]

where v(t) is the signed velocity, w(t) is the load ratio, and a(t) is the acceleration. The load ratio is normalised into a percentage relative to the rated load, as described in Equation (5) and Table 1.

The network outputs are the predicted voltage and driving force, thereby establishing the mapping relationship between the electrical system and the dynamic system:

(10) $$y\left(t\right)=[V_{pred}\left(t\right),\;F_{pred}\left(t\right)]$$

That is, the model simultaneously predicts the voltage response and the dynamic driving force.

In conventional PINN frameworks, system deviation is often represented by a single residual, for example:

(11) $$R\left(t\right)=y_{pred}\left(t\right)-y_{meas}\left(t\right)$$

Training based only on a single output error is sensitive to measurement noise and makes it difficult to distinguish electrical abnormalities from mechanical faults. To improve the robustness of parameter identification, this study constructs both voltage residuals and driving force residuals, so that the model is constrained from two physical domains:

(12) $$R_{v}\left(t\right)=V_{pred}\left(t\right)-V\left(t\right)$$

(13) $$R_{f}\left(t\right)=F_{pred}\left(t\right)-F_{trac}\left(t\right)$$

where

(14) $$F_{trac}\left(t\right)=\left(V\left(t\right)-V_{0}-\left|v\left(t\right)\right|\kappa \left(v\right)\right)K_{IR}$$

In practical traction elevator systems, upward and downward motions often exhibit asymmetric electrical behaviour due to differences in motor driving, gravitational assistance, and braking mechanisms. In particular, conventional elevator systems without fully regenerative operation may rely more heavily on braking mechanisms during downward motion. To represent this asymmetry, a directional response function κ(v) is introduced:

(15) $$\kappa \left(v\right)=\left\{\begin{array}{cc} K_{up}, & v>0\\ K_{down}, & v<0 \end{array}\right.$$

where $K_{up}$ and $K_{down}$ denote the equivalent electrical response coefficients during upward and downward motion, respectively.

The voltage residual reflects deviations in the motor electrical system, whereas the driving force residual represents deviations in the dynamic system. Since these two residuals originate from different physical pathways, they improve model robustness and enhance anomaly detection capability. Although the two residuals are mathematically related to some extent, their different physical origins may lead to distinct characteristics under different operating conditions, thereby improving detection reliability.

The training objective of PINN is to minimise the combined loss function:

(16) $$L_{total}=\lambda_{1}L_{volt}+\lambda_{2}L_{force}+\lambda_{3}L_{prior}$$

where $\lambda_{1}$, $\lambda_{2}$, and $\lambda_{3}$ are weighting coefficients for the three loss terms. $L_{volt}$ is the voltage residual loss used to evaluate deviations in electrical signals, $L_{force}$ is the force residual loss used to evaluate deviations in mechanical signals, and $L_{prior}$ is the prior constraint loss used to restrict parameters within reasonable ranges. The three loss terms are defined as follows:

(17) $$L_{volt}=\frac{1}{N}\sum {\left(V_{pred}-V\right)}^{2}$$

(18) $$L_{force}=\frac{1}{N}\sum {\left(F_{pred}-F_{trac}\right)}^{2}$$

(19) $$L_{prior}=\sum {\left(\frac{\theta -\theta_{0}}{\sigma }\right)}^{2}$$

The parameters to be learned in PINN include:

(20) $$\theta =\{M_{car},\mu ,K_{IR},K_{e}\}$$

To ensure physical plausibility, nonlinear mappings are adopted to guarantee θ>0, thereby avoiding nonphysical solutions during training, such as negative mass or negative damping.

During training, the PINN model performs parameter identification by minimising the combined loss function. This function includes voltage residual loss, dynamic residual loss, and prior parameter constraint loss. By jointly considering data fitting errors and physical constraints, PINN can learn system parameters with physical meaning. Compared with conventional PINN approaches, the proposed model is formulated based on engineering input–output relationships and incorporates dual-residual constraints together with prior parameter limits, thereby improving model stability and reducing computational complexity.

The residual neural network and the physical parameters are optimised simultaneously during training through backpropagation, allowing the model to maintain physical consistency while compensating for modelling uncertainties.

## 4. PINN-Based Elevator Health Assessment and Degradation Warning

### 4.1. Data Preprocessing and Operating State Feature Construction

As field sensors are usually deployed independently, different signals often have inconsistencies in sampling frequency, timestamps, and data formats. Therefore, raw data cannot be directly used for model computation. Before data analysis, multi-source sensor data must first undergo time alignment and feature reconstruction so that the original measurements can be converted into unified sequential input data.

In practical elevator systems, commonly available signals include those from speed sensors or encoders, load sensors, voltage and current sensors, as well as discrete control signals such as door status and travel direction. These data exhibit typical multi-source heterogeneous characteristics. On one hand, different sensors often operate with different sampling intervals and timestamps. On the other hand, the data contain both continuous variables and discrete state signals. In addition, the physical meanings of these raw signals are not uniform, making it difficult to directly represent system dynamics. For example, speed is a continuously varying signal, whereas door status is a discrete logic variable. Therefore, data alignment is required through resampling, interpolation, and state synchronisation under a unified time step, followed by the construction of feature variables that can characterise elevator operating conditions.

Such issues are common in industrial Internet of Things applications and equipment monitoring, where unified sequential inputs are typically established through data alignment and feature reconstruction [19]. In this study, the sampling frequency is relatively low, approximately 1 Hz. Under such conditions, acceleration obtained directly from speed differencing is highly sensitive to noise, especially during starting and stopping stages, which can significantly affect dynamic residual calculations. To improve robustness, a dual-path acceleration estimation method is proposed.

The first path estimates acceleration from speed variation:

(21) $$a_{A}\left(t\right)=\frac{v(t)-v(t-1)}{\Delta t}$$

This method responds quickly and can capture transient dynamics, but it is sensitive to noise and strongly dependent on sampling frequency. Under low-rate industrial data, it may produce large errors and false acceleration spikes during start-up and braking stages [20]. Since acceleration directly enters the dynamic calculation F=m·a, such errors may be amplified and reduce the accuracy of force residuals. Therefore, smoothing is applied during preprocessing to suppress noise effects.

The second path provides a more robust estimate under steady operating conditions by inferring acceleration from load variation. When the elevator door is closed, passenger loading is assumed to remain constant. Under this condition, variations in the measured car load are primarily caused by inertial effects during acceleration and deceleration. Therefore, the change in measured load, denoted as ΔW(t), can be used as an indirect indicator of elevator acceleration. The coefficient $K_{a}$ represents an equivalent conversion factor between load variation and acceleration and can be determined empirically from historical operating data.

(22) $$a_{B}\left(t\right)=\frac{\Delta W(t)}{K_{a}}$$

This method has stronger noise resistance, but it relies on door status signals and the assumption of constant cabin load.

To combine the advantages of both approaches, weighted fusion is adopted:

(23) $$a\left(t\right)=\alpha a_{A}\left(t\right)+\left(1-\alpha \right)a_{B}\left(t\right)$$

where α is the weighting factor of the speed-differencing estimate $a_{A}$. Operating-condition constraints are further introduced. A larger weight is assigned to $a_{A}$ during starting and braking stages, while a larger weight is assigned to $a_{B}$ during steady travel.

This acceleration estimation strategy is essentially a multi-source information fusion method. It offers improved robustness, stronger noise resistance, and suitability for low-sampling-rate systems, making it particularly appropriate for the present elevator operation case.

### 4.2. Multi-Residual Health Indicators and Risk Evolution Model

Based on the trained PINN parameters, the elevator system can continuously compute the voltage residual $R_{v}\left(t\right)$ and force residual $R_{f}\left(t\right)$. However, these two residuals originate from different physical domains and therefore differ significantly in units and numerical scale. For example, the voltage residual is usually measured in volts, whereas the force residual is related to equivalent system mass and acceleration, and its magnitude may be several or even tens of times larger. If a combined residual is constructed directly, the larger residual term may dominate the result and weaken the contribution of the other physical domain.

To avoid this issue, normalisation is applied so that both residuals are transformed into a dimensionless space:

(24) $${\tilde{R}}_{v}\left(t\right)=\frac{R_{v}\left(t\right)}{\sigma_{v}},\;{\tilde{R}}_{f}\left(t\right)=\frac{R_{f}\left(t\right)}{\sigma_{f}}$$

where $\sigma_{v}$ and $\sigma_{f}$ are the statistical scales of the voltage and force residuals, such as standard deviations or rated values. A combined residual is then defined as:

(25) $$R\left(t\right)=\sqrt{{\tilde{R}}_{v}^{2}\left(t\right)+{\tilde{R}}_{f}^{2}\left(t\right)}$$

This treatment ensures that electrical and mechanical residuals contribute equally to the health indicator, thereby improving diagnostic completeness and robustness.

Traditional anomaly detection methods usually rely on fixed thresholds or moving averages. Such approaches are sensitive to transient noise and often fail to distinguish short disturbances from persistent abnormalities, leading to relatively high false alarm rates in industrial systems. To overcome this limitation, a residual time-accumulation method is introduced by jointly considering residual magnitude and persistence duration. Similar to health index modelling in predictive maintenance, the residual is regarded as an indicator of deviation from normal operation, and a risk index is constructed through temporal accumulation.

To prevent extreme outliers from dominating risk evaluation, nonlinear compression is first applied to the residual:

(26) $$R_{s}\left(t\right)=R_{max}\tanh\left(\frac{R(t)}{R_{max}}\right)$$

This operation suppresses abnormal peaks while preserving the overall trend.

After nonlinear compression, a sliding-window statistical method is used to estimate the residual distribution. The moving mean $\bar{R}\left(t\right)$ and standard deviation $\sigma_{R}\left(t\right)$ are calculated, and an adaptive abnormal boundary is defined according to the three-sigma rule:

(27) $$R_{th}\left(t\right)=\bar{R}\left(t\right)+3\sigma_{R}\left(t\right)$$

The excessive residual above the threshold is then defined as:

(28) $$R_{e}\left(t\right)=\max\left(R_{s}\left(t\right)-R_{th}\left(t\right),0\right)$$

Compared with a fixed threshold, this adaptive strategy can better accommodate changing operating conditions while filtering occasional noise or outliers.

To characterise abnormal persistence, the duration of continuous anomalies is further introduced as:

(29) $$D\left(t\right)=\left\{\begin{array}{cc} D(t-1)+1, & R_{e}\left(t\right)>0\\ 0, & \mathrm{otherwise} \end{array}\right.$$

This variable describes the temporal continuity of abnormal conditions. In engineering systems, short anomalies are often caused by transient disturbances or sensor noise, whereas persistent anomalies are more likely to indicate degradation or latent faults, such as lubrication deterioration, guide rail wear, or reduced motor efficiency.

Based on residual magnitude and duration, a nonlinear time-dependent risk model is established:

(30) $$Risk\left(t\right)=\lambda Risk\left(t-1\right)+R_{e}\left(t\right)e^{\beta D(t)}$$

where λ represents the temporal decay factor controlling the fading memory of past anomalies, while β controls the amplification effect of anomaly duration. The absolute values of Risk(t) are relative health indicators rather than direct physical quantities and are mainly used for trend analysis and maintenance decision support. When the system returns to normal, the risk value gradually decreases. When anomalies persist, the risk grows exponentially. Compared with conventional linear accumulation methods, this nonlinear model is more sensitive to progressive abnormalities while suppressing misjudgment caused by transient disturbances.

Using the above framework, health indicators can be updated in real time based on refreshed PINN parameters, and system degradation trends can be continuously monitored. When the risk index keeps increasing, potential fault risks can be identified in advance and warning actions can be triggered. Compared with conventional threshold-based methods, the proposed approach can reduce false alarms and provide earlier fault detection, making it suitable for predictive maintenance and intelligent operation of elevator systems.

In addition, the residual-based risk modelling framework has good scalability. Different degradation scenarios can be identified through different residual features, for example by incorporating travel direction, load variation, or acceleration characteristics. The method does not require predefined fault labels or specific fault mechanism models, which gives it strong transferability.

### 4.3. Deployment Implementation and System Architecture

**Figure 3:**
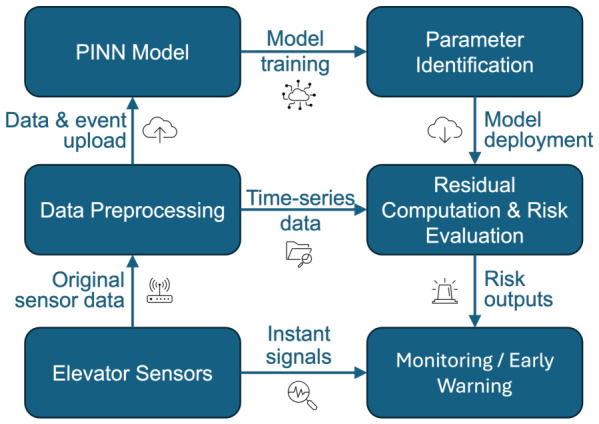
Overall framework of the elevator health monitoring and warning system.

To meet the practical requirements of real-time monitoring and long-term model updating for elevator systems, a hierarchical deployment framework for field applications is proposed in this study, as shown in Figure 3. The framework consists of a data acquisition layer, an online analysis layer, and a model management layer. These functional modules operate collaboratively and form a closed-loop feedback mechanism.

The data acquisition layer is mainly responsible for real-time sensing of elevator operating conditions and basic safety protection. Sensors deployed in the control cabinet and inside the elevator car collect raw operational signals such as speed, voltage, load, floor position, and door status. These signals directly reflect the operating state of the elevator and provide the data foundation for subsequent health assessment. Since field control devices are usually embedded in the elevator control system, their computational resources are limited but their response speed is high. Therefore, this layer still adopts rule-based logic or threshold strategies for rapid protection. When obvious abnormalities are detected, alarms, speed limitation, or emergency shutdown actions can be triggered immediately to ensure safe operation [21].

The online analysis layer mainly performs data preprocessing, state estimation, and risk assessment. Since raw data originate from different sensors, inconsistencies in sampling frequency, timestamps, and data formats are common. Therefore, time alignment and feature reconstruction are first carried out to generate unified sequential input variables. Based on these inputs, the trained PINN model is used for parameter estimation, dual-residual calculation, and health indicator updating, enabling low-latency condition monitoring and degradation risk warning. This layer is deployed close to the data source and can satisfy the practical requirements of real-time response and continuous monitoring [22,23].

The model management layer is responsible for computation-intensive tasks such as model training, parameter updating, and historical data analysis. By combining long-term operational data with physical constraints, key parameters of the elevator system can be periodically identified and the PINN model can be continuously optimised. The updated model parameters are then redeployed to the online analysis layer for subsequent real-time inference and health assessment. This deployment strategy of offline training and online inference balances model accuracy, computational efficiency, and long-term adaptability [24,25].

In addition, the risk indicators generated by the online analysis layer can be fed back to the field control logic to dynamically adjust alarm thresholds or support maintenance decisions. In this way, a closed-loop operating mechanism of data acquisition, state analysis, model updating, risk warning, and safety protection is established. The proposed architecture has strong engineering practicality, reduces communication burden while maintaining real-time performance, and supports long-term predictive maintenance requirements.

## 5. Results Analyses

### 5.1. Experimental Setup and Dataset

To verify the effectiveness of the proposed elevator health monitoring and degradation risk warning method, experiments were conducted using real operational data from an elevator system. The test object was a traction passenger elevator, model GRPS30 manufactured by Sicher Elevator Co., Ltd., Huzhou, China, with a rated load of 1000 kg and a rated speed of 1.75 m/s. The elevator serves 26 floors with 26 stops and 26 doors. This type of elevator is a typical medium-speed passenger system widely used in high-rise buildings and therefore has strong engineering representativeness.

The experimental data were collected directly from the elevator control cabinet. The recorded signals included operating speed, voltage, cabin load, door status, travel direction, and floor information, together with other multi-source sensor data. These measurements reflect both elevator operating conditions and drive system behaviour, providing the basis for subsequent physics-based degradation analysis. The sampling frequency was 1 Hz, meaning one record per second, which is consistent with the typical configuration of industrial remote elevator monitoring systems and therefore has practical relevance.

A total of 3 h of continuous operational data, corresponding to approximately 10,800 samples at a sampling frequency of 1 Hz, were collected from the elevator control cabinet. During this period, the elevator responded to 80 upward and downward service calls across different floors. The dataset covers multiple representative operating conditions, including no-load travel, partial-load travel, and frequent start–stop operation. These operating scenarios provide diverse dynamic characteristics for PINN training and validation.

It should be noted that elevator operation is inherently intermittent. Although the complete dataset contains 10,800 samples, not all samples contribute equally to the parameter identification process. The proposed PINN is derived from the elevator dynamic and motor models and is therefore primarily informative during active motion. Using a velocity threshold of 0.05 m/s, approximately 957 samples (8.9% of the dataset) correspond to elevator movement. These samples are distributed across approximately 80 independent travel events involving different travel distances, directions, and load conditions.

The remaining samples mainly represent stationary conditions, including passenger waiting periods, door opening and closing operations, and floor holding states. During these periods, elevator behaviour is dominated by control logic, braking mechanisms, and safety interlocks rather than the dynamic relationships described by the proposed model. To prevent the optimisation process from being dominated by these highly correlated stationary samples, a motion-state weighting strategy was adopted during training, assigning substantially higher weights to moving samples while retaining stationary samples to preserve overall operational consistency.

In addition, since the data were collected from a normally operating elevator, no obvious fault samples were included. This is consistent with practical maintenance scenarios, where degradation warning and condition assessment must often be achieved under limited or unavailable fault data conditions.

All experiments, simulations, and model training were implemented in the Python 3.13.5. The PINN model was developed using PyTorch 2.10.0, together with NumPy 2.4.2 and SciPy 1.17.0 for data processing, numerical computation, and signal analysis. The training stage was mainly used for parameter identification and network optimisation, while the online inference stage was used for real-time residual calculation, health indicator updating, and risk assessment, thereby simulating the practical workflow of offline training and online deployment.

The experimental tasks mainly consisted of three parts. First, the elevator data were processed and used to train the PINN model, identify key physical parameters, and establish the mapping relationship between the elevator dynamic model and the motor model. Second, the trained model parameters were used in the inference stage to calculate voltage residuals, dynamic residuals, and time-accumulated risk indicators. Third, different degradation scenarios were constructed to evaluate the ability of the proposed method to detect progressive abnormalities, including motor degradation, braking system degradation, and increased friction.

Through the above experimental setup, the effectiveness of the proposed framework can be systematically validated under real elevator operational data, and the variation characteristics of risk indicators under different degradation scenarios can be analysed, providing the basis for the following results and discussion.

### 5.2. PINN Parameter Identification and Dual-Residual Analysis

This section validates the proposed PINN-based elevator health monitoring framework using real operational data. The experimental results presented in this subsection focus on data preprocessing, PINN parameter identification, and dual-residual analysis. The degradation risk warning performance is evaluated separately in the following subsection. The objective is to demonstrate the feasibility and effectiveness of the proposed method under practical operating conditions.

**Figure 4:**
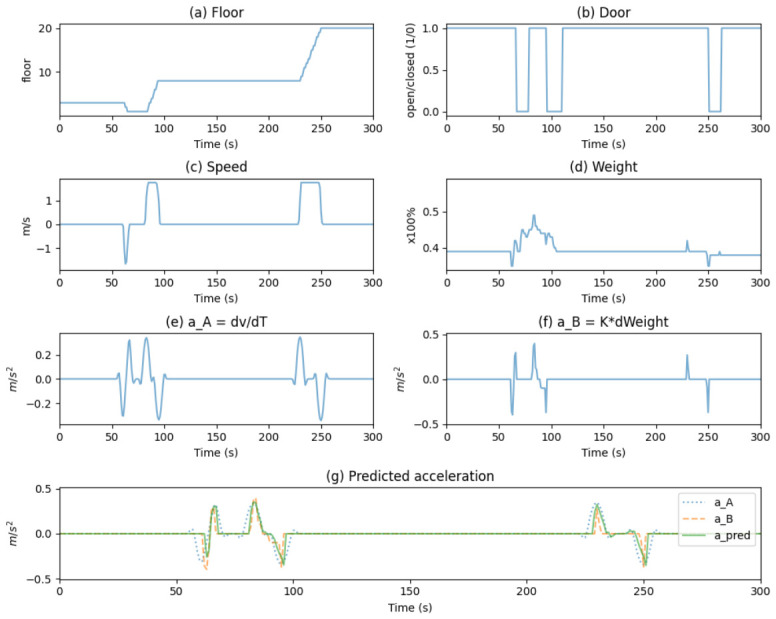
Results of data preprocessing and dual-path acceleration estimation.

Figure 4 presents the preprocessing results obtained using the method described in Section 4.1. The aligned multi-source operational data, including floor position, speed, load, and door status, provide a consistent time-series representation of the elevator operating state. Based on the proposed dual-path acceleration estimation framework, the acceleration profile is reconstructed from both speed information and load variation information.

As observed in Figure 4g, the two acceleration estimation paths exhibit complementary characteristics. The speed-differentiation path provides a fast response to acceleration and deceleration events but is more sensitive to measurement noise and low-frequency sampling effects. In contrast, the load-based path produces a smoother acceleration estimate and is less affected by signal fluctuations, although it may exhibit slower dynamic response under certain operating conditions. In this study, a fixed weighting coefficient of α=0.7 was adopted for the fusion process, giving greater emphasis to the speed-based estimate while retaining the robustness of the load-based estimate. This value was selected empirically for the investigated dataset and reflects a compromise between dynamic responsiveness and noise suppression.

It should be noted that α is not a fixed parameter of the proposed framework. In practical deployments, the weighting coefficient may be adjusted according to sensor quality, operating conditions, or signal confidence. For example, when speed measurements become noisy or unreliable, a lower value of α can be adopted to increase reliance on the load-based estimate. Conversely, when load measurements are affected by disturbances or sensor uncertainty, a higher value of α may be preferred. Therefore, the proposed formulation provides flexibility for future adaptive weighting strategies.

The fused acceleration signal shown in Figure 4g preserves the major dynamic characteristics of elevator motion while suppressing fluctuations caused by low sampling frequency and measurement noise. These results demonstrate that the proposed preprocessing framework can effectively transform heterogeneous field sensor data into physically meaningful state variables suitable for subsequent PINN parameter identification and residual generation.

**Figure 5:**
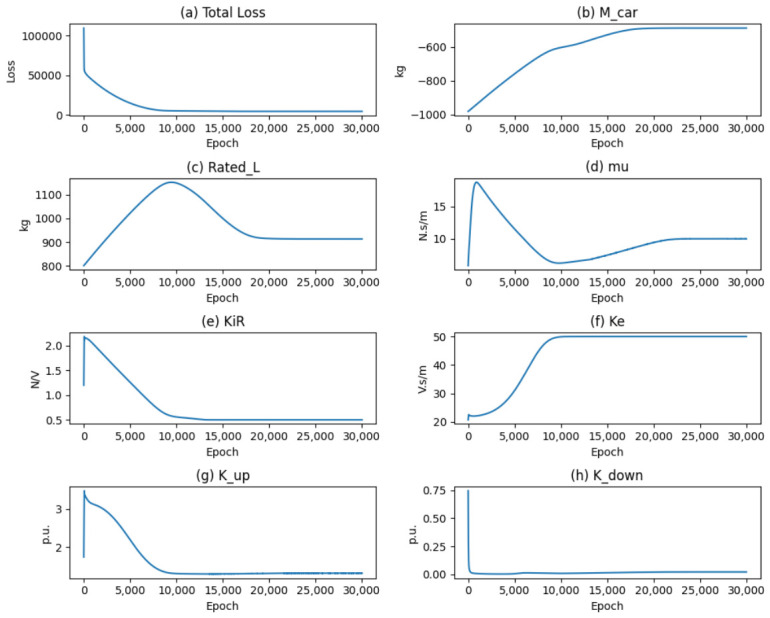
PINN training convergence process and parameter identification results.

Figure 5 presents the training process of the PINN model and the convergence results of key physical parameters. It can be observed that the total loss function decreases rapidly during the early training stage and then gradually converges to a stable value, indicating that the proposed model can effectively learn the operating characteristics and hidden parameters of the elevator system.

During training, the total loss decreases from an initial magnitude of approximately $10^{5}$ to about $10^{3}$ in the stable stage, corresponding to an overall reduction of more than two orders of magnitude. This indicates that the model can effectively learn the physical characteristics of the elevator system. The key parameters gradually converge after approximately 20,000 training epochs, demonstrating good convergence behaviour and training stability.

Compared with conventional purely data-driven methods, an important advantage of PINN is that the identified parameters have clear physical meaning. For example, the equivalent mass parameter can reflect load balance conditions, the friction coefficient can indicate mechanical wear or lubrication status, and the drive parameters are related to motor performance. Therefore, these parameters can be used not only for predictive modelling but also as interpretable indicators of equipment health condition.

**Figure 6:**
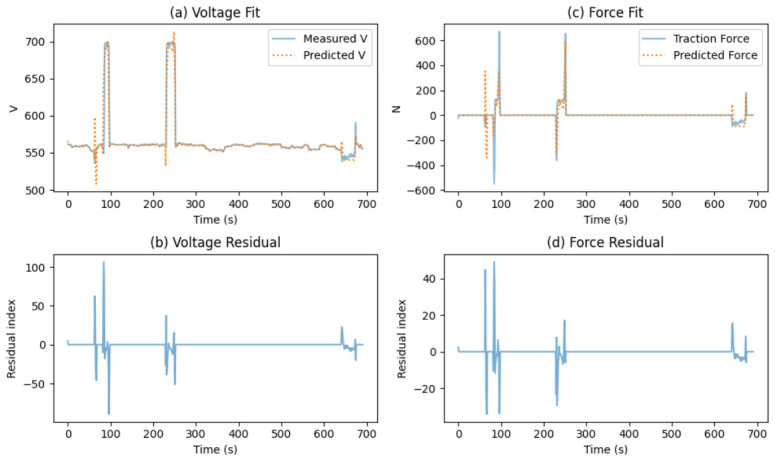
PINN prediction results and dual-residual analysis.

Figure 6 further presents the model prediction results together with the corresponding dual-residual analysis. The results present a representative 700 s excerpt selected from the complete operational dataset for visualisation purposes. The excerpt was randomly chosen from the normal operating data and includes multiple elevator acceleration, cruising, and deceleration events, thereby providing a representative illustration of the model fitting and residual generation performance. The results show that the predicted voltage is highly consistent with the measured voltage trend, while the estimated driving force can also effectively reflect the system operating condition. This demonstrates that the established dynamic model and PINN framework provide good fitting capability.

It can be observed that both residuals remain close to zero for most operating periods, indicating that the proposed PINN model provides consistent predictions for both electrical and dynamic quantities under normal operating conditions. Larger residual peaks mainly occur during acceleration and deceleration stages, where rapid state transitions introduce greater modelling uncertainty.

Although the voltage residual and force residual exhibit similar temporal patterns, this behaviour is expected because both residuals originate from the same elevator operating process and are physically related through the electromechanical model. Therefore, the objective of the proposed dual-residual framework is not to create two completely independent fault indicators. Instead, the two residuals provide complementary consistency checks from different physical domains. The voltage residual evaluates the consistency of the electrical drive behaviour, whereas the force residual evaluates the consistency of the dynamic force balance derived from the elevator motion model.

The value of the dual-residual design lies in improving the robustness and interpretability of condition assessment. When both residuals remain small, the electrical and mechanical behaviours are simultaneously consistent with the identified physical model. Conversely, when residual deviations emerge, the two residuals provide multiple physical perspectives for evaluating system consistency, thereby reducing the risk of relying on a single measurement pathway. This dual-domain validation mechanism forms the basis for the subsequent health indicator construction and degradation risk assessment framework.

### 5.3. PINN-Based Risk Assessment and Early Warning

To verify the degradation warning capability of the proposed method, three typical progressive degradation scenarios are constructed in this study, including reduced motor drive performance, braking performance deterioration, and increased mechanical friction. In the early stage, these problems are usually manifested only as efficiency reduction, increased operational fluctuation, or higher energy consumption, making them difficult to identify in time using conventional threshold-based methods. Therefore, they are suitable test cases for early warning evaluation.

**Figure 7:**
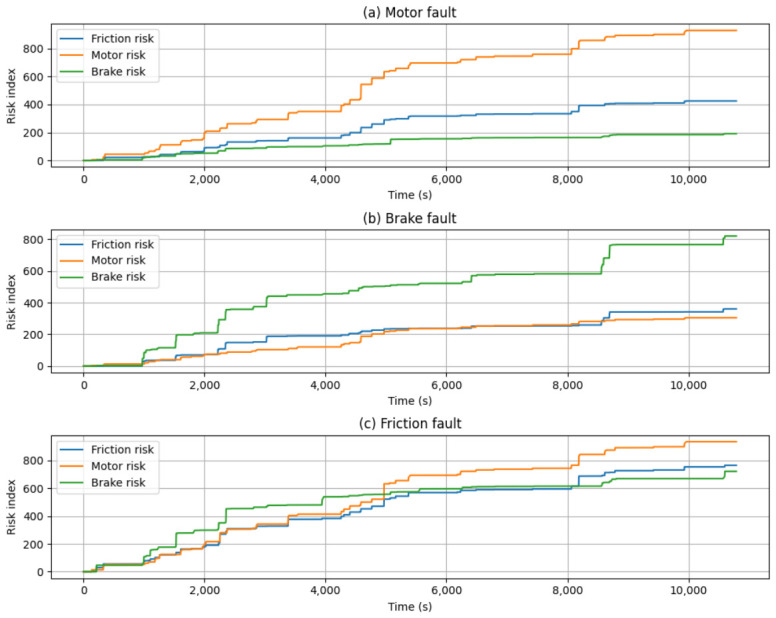
Risk assessment results under different degradation scenarios.

Figure 7 shows the evolution results of the risk index under different degradation scenarios. Unlike conventional detection methods based on instantaneous thresholds, the proposed approach employs a time-accumulated risk model that jointly considers abnormal magnitude and persistence duration, enabling the system to distinguish short-term disturbances from sustained degradation trends.

In the motor degradation scenario, the motor-related risk increases significantly, while the other indicators remain at relatively low levels, indicating that the proposed method can effectively identify abnormalities in the drive system. In the braking degradation scenario, the braking-related risk rises markedly, whereas the remaining indicators show only minor changes, further verifying the capability of the model to distinguish different degradation modes.

In the friction degradation scenario, multiple risk indicators increase simultaneously, suggesting that friction changes can affect the overall operating condition of the system. This observation is consistent with practical engineering experience, where guide rail wear or insufficient lubrication often leads to increased energy consumption, reduced ride smoothness, and higher driving load.

From the quantitative results, clear differences in risk values can be observed among the degradation scenarios. For example, under the motor degradation condition, the corresponding risk value exceeds 900, while the other indicators remain below 450. Under the braking degradation condition, the braking-related risk exceeds 800, whereas the remaining indicators stay below 400. These results demonstrate that the proposed method can effectively distinguish different types of degradation problems.

In addition, the time-accumulated risk model can significantly reduce false alarms caused by transient noise. During normal operation, the risk index remains stable even when short-term residual peaks occur. When abnormalities persist, however, the risk gradually accumulates and can trigger warnings even if the magnitude of each individual residual is relatively small. This mechanism is more consistent with the actual evolution process of equipment degradation and is more suitable for predictive maintenance of safety-critical systems such as elevators.

It should be noted that the proposed Risk(t) indicator is not intended to directly replace the existing safety protection logic of elevator systems. Instead, it serves as a degradation-oriented health indicator for maintenance decision support and long-term condition monitoring. In practical applications, moderate and slowly increasing Risk(t) values may indicate early-stage degradation and can be used to trigger maintenance recommendations or increase inspection frequency. In contrast, rapidly increasing or persistently high Risk(t) values may indicate severe deterioration requiring operational intervention or temporary service suspension.

Therefore, the proposed temporal risk model provides a continuous health evaluation mechanism between ’fully normal operation’ and ’hard safety shutdown’, enabling maintenance personnel to observe degradation trends before critical faults occur. This characteristic is particularly important for predictive maintenance applications, where the objective is not only to detect faults after threshold violations, but also to identify gradual degradation at an earlier stage while minimising unnecessary false alarms.

### 5.4. Comparison with Conventional Threshold-Based Monitoring

**Figure 8:**
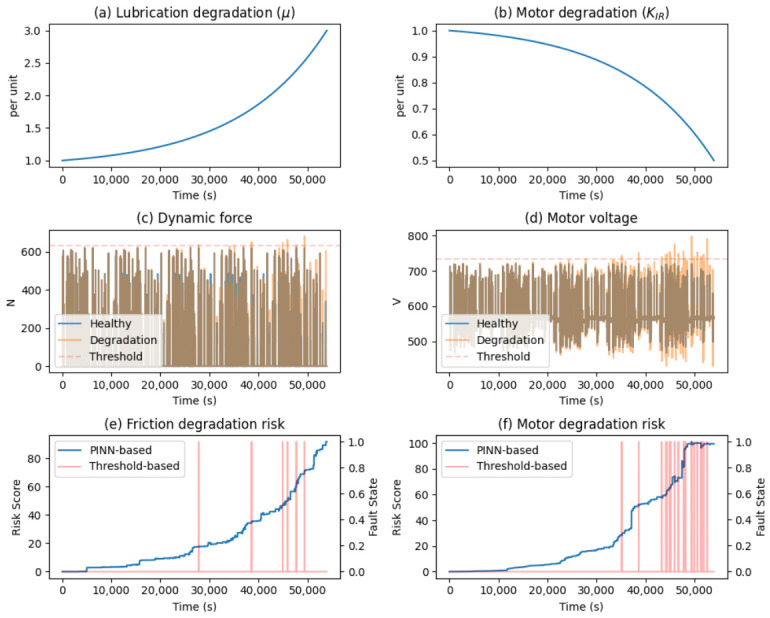
Early warning comparison between the PINN-based degradation index approach and the threshold-based fault status approach.

Figure 8 compares the conventional threshold-based method with the proposed PINN-based risk assessment framework under two gradual degradation scenarios, including lubrication degradation represented by an increasing friction coefficient μ and motor degradation represented by a decreasing motor driving coefficient $K_{IR}$. Unlike abrupt failures, these faults evolve progressively over time and usually exhibit only weak deviations during the early stage, making them suitable for evaluating predictive maintenance and early warning capability.

As shown in Figure 8a,b, both degradation parameters vary gradually with time. The lubrication degradation scenario is modelled by a progressive increase in the friction coefficient, while the motor degradation scenario is represented by a gradual reduction in motor driving capability. Such degradation profiles better reflect real industrial ageing processes, in which system performance deteriorates progressively rather than failing suddenly.

Figure 8c,d illustrate the monitoring results of the conventional threshold-based method. In the lubrication degradation case, the monitoring is performed using a friction force threshold, while the motor degradation case relies on a voltage threshold. It can be observed that during the early degradation stage, although the system behaviour has already deviated from the healthy condition, the signal magnitude still remains below the predefined threshold. Consequently, the conventional method fails to produce an alarm until the degradation becomes sufficiently severe to cause large instantaneous signal deviations. Furthermore, due to operating-condition variations and measurement fluctuations, the threshold-based method exhibits intermittent alarm spikes. This phenomenon can be observed in Figure 8e,f, where the red alarm-state signals contain multiple isolated peaks, indicating that the conventional method is sensitive to transient disturbances and measurement noise.

In contrast, the proposed PINN-based method does not rely solely on single-signal threshold monitoring. Instead, it evaluates the physical consistency among multiple variables through the PINN model and computes temporal risk indicators based on residual evolution. The key difference is that the threshold-based approach only determines whether a signal exceeds a predefined limit, whereas the proposed method evaluates whether the overall physical behaviour of the elevator continuously deviates from the learned normal operating condition. Therefore, even when individual sensor signals remain within acceptable limits, persistent small deviations can still accumulate into an increasing degradation risk.

This characteristic is clearly demonstrated in Figure 8e,f. The blue curves representing the PINN-based risk indicators begin to increase gradually during the early stage of degradation and continue to rise as the degradation progresses. In comparison, the threshold-based method only generates frequent alarms during the later stage of degradation. In particular, under the lubrication degradation scenario, the PINN-based risk starts to exhibit a noticeable upward trend at approximately $1.5\times 10^{4}$ s, whereas the threshold-based alarms do not become frequent until approximately $2.8\times 10^{4}$ s. This result demonstrates that the proposed PINN-based framework is capable of identifying degradation trends significantly earlier than conventional threshold-based monitoring methods.

Another important distinction lies in the representation of system health. Conventional threshold-based approaches produce binary alarm outputs, indicating only whether a threshold has been exceeded. In contrast, the proposed method generates a continuous health indicator, which reflects both the severity and temporal evolution of system degradation. Such continuous risk representation is more suitable for predictive maintenance applications. For example, in Figure 8f, the motor degradation risk increases progressively over time, enabling maintenance personnel to observe the degradation trend and schedule maintenance before safety-critical faults occur.

In the investigated degradation scenarios, the threshold-based method generated more than 6 isolated alarm activations in lubrication degradation and more than 16 alarm activations in motor degradation before a persistent fault trend became evident. In contrast, the proposed PINN-based framework produced a continuously evolving risk indicator without repeated alarm oscillations, thereby significantly reducing nuisance alarm events caused by transient fluctuations.

Overall, the results indicate that conventional threshold-based monitoring is more suitable for detecting abrupt faults, whereas its capability for gradual degradation detection is limited and susceptible to transient disturbances. By combining physics-informed residual modelling with temporal risk accumulation, the proposed PINN-based framework can identify degradation trends at an earlier stage while significantly reducing false alarms caused by temporary fluctuations. Therefore, the proposed method is more suitable for long-term elevator health monitoring and predictive maintenance applications.

## 6. Conclusions

This paper proposed a PINN-based method for elevator health monitoring and degradation risk warning. To address the limitations of conventional data-driven approaches, including weak physical interpretability and strong dependence on fault samples, the elevator dynamic model and traction drive model were embedded into the neural network training process, enabling online identification of key hidden parameters and system condition assessment. First, to handle practical data issues such as multi-source heterogeneity, asynchronous sampling, and low sampling frequency, multi-sensor data were processed through time alignment and feature reconstruction. A dual-path acceleration estimation method was further developed, which improved the stability and accuracy of dynamic state estimation under low-frequency data conditions. Second, a PINN model for traction elevator systems was established by integrating operational data with physical constraints, allowing identification of key parameters such as equivalent mass, friction coefficient, and drive coefficient. Furthermore, dual-residual health indicators based on electrical residuals and dynamic residuals were constructed to characterise system operating conditions from different physical perspectives. Finally, a time-accumulated risk model considering abnormal magnitude and persistence duration was proposed to achieve early detection and warning of progressive degradation faults. Experimental results showed that the proposed method achieved stable parameter convergence and effectively identified abnormal system conditions. The constructed risk indicators were able to reveal degradation trends significantly earlier than conventional threshold-based monitoring methods while reducing nuisance alarms caused by transient disturbances and measurement noise. The dual-residual mechanism further improved the robustness of anomaly detection and enhanced the physical interpretability of diagnostic results. Overall, this study demonstrated the application potential of PINN in elevator health monitoring and provided a practical solution for intelligent maintenance of conventional electromechanical equipment under limited fault data conditions, particularly for early degradation detection and predictive maintenance applications. Future work will incorporate longer-term field operational data to further verify model generalisation capability and explore online incremental learning, adaptive parameter updating, and extension to other vertical transportation systems.

## Figures and Tables

**Table 1:** Available operational signals of the traction elevator from the control cabinet.

Signal Variable	Type/Unit	Description
Floor call	Floor level (integer)	Selected destination floor
Floor position	Floor level (integer)	Current floor of the elevator
Levelling status	Discrete state	Levelled/not levelled
Door status	Discrete state	Opening/open/closing/closed
Travel direction	Discrete state	Upward/downward/stopped
Travel speed	m/s	Real-time speed of the car
Command speed	m/s	Target speed from the controller
Passenger load	%	Percentage of rated load
Drive voltage	V	Input voltage of the traction machine
Heat sink temperature	°C	Temperature of the drive unit

## Data Availability

The data presented in this study are available on request from the corresponding author.
